# Surgical resident satisfaction with the current surgical training program in the Riyadh area

**DOI:** 10.4103/0256-4947.55170

**Published:** 2009

**Authors:** Saud Al Shanafey, Ali Alzahrani, Abdulrahman AlBallaa, Abdulaziz Alballaa

**Affiliations:** a From the Department of Surgery, King Faisal Specialist Hospital and Research Centre, Riyadh, Saudi Arabia; b From the Departments of Biostatistics, Epidemiology and Scientific Computing, King Faisal Specialist Hospital and Research Centre, Riyadh, Saudi Arabia; c From the Department of Surgery, King Abdulaziz Medical City, Riyadh, Saudi Arabia; d From the Department of Surgery, King Khalid Hospital, Riyadh, Saudi Arabia

## Abstract

**BACKGROUND:**

The satisfaction of surgical residents with their training programs plays an important role in dictating its output. This survey was conducted to explore the satisfaction of surgical residents with their training programs in the Riyadh area.

**METHODS:**

A survey questionnaire was distributed in four major hospitals to explore the view of surgical residents regarding their training programs. Frequency tables were generated for each question in the survey.

**RESULTS:**

About 78 survey forms were distributed and 52 were retrieved (67%). About 45% of residents had a comprehensive orientation on admission to the program, but only 20% felt it was helpful. Only 40% of residents felt that their trainers were committed to training and that the consultants who were trained abroad were more committed than those trained locally (62% *vs* 36%, *P*=.01). Only 15% felt the residents themselves had enough bedside teaching or operative experience. Seventy-eight percent of the residents felt that current training does not meet their expectations. However, 85% felt that training abroad was better than local training, and 60% felt it should be mandatory. While 90% felt that training programs should be unified nationally and controlled by one organization, only 6% felt that the current governing body was capable of monitoring the training. Moreover, only 28% felt that current reviews of programs by the governing body are effective.

**CONCLUSIONS:**

These results show that surgical residents are generally dissatisfied with current training programs. The study suggests that there are significant weaknesses in the current programs and the governing body may be ineffective in monitoring the programs. We feel that a national review of surgical training programs is warranted in view of these results.

Satisfaction of trainees with their training program is one of the most important factors affecting its output. This has been shown in other health care training fields, and found to be an indicator of quality processes.^1^ Health care in Saudi Arabia enjoys wide support from many government sectors with multiple health care systems operating many hospitals with varying levels of care. Surgical training in Saudi Arabia has gone through some phases of change involving the authority charged with monitoring the training programs. While it is understood that universities are responsible for training residents in many countries, currently the Saudi Commission for Health Specialties (a government organization) oversees all aspects of training. These include program design, training center accreditation, resident selection, the course and final exams, and also physician certification and licensing. The satisfaction of Saudi surgical residents with their training programs has not been assessed. This survey was conducted to explore the satisfaction of surgical residents with their training program in the Riyadh area.

## METHODS

A survey questionnaire was designed to explore the view of surgical residents regarding many aspects of their training programs, which included program orientation, mentoring, faculty role, hospital role, academic activities, clinical activities including operative experience, evaluation process, external training and training policies and monitoring. Two epidemiologists (among the authors of the manuscript) had designed the questionnaire, and a few clinicians were consulted to review and suggest any modifications. The survey contained direct questions with two responses: yes and no. We elected to design the survey with direct questions after consulting with a few professionals in the field who were interested in the subject. We also wanted to avoid any gray-zone opinions which could render the survey results equivocal. In addition, we believed that direct questions with few answer options improve response rates. The survey was distributed to all surgical residents rotating in four major hospitals in the Riyadh area between July 2007 and January 2008. These included King Faisal Specialist Hospital and Research Center, King Abdulaziz Medical City, King Khalid University Hospital, and The Armed Forces Hospital. These hospitals were chosen due to their reputable established surgical programs and the presence of a relatively high number of trainees among their staff. Concerned program directors were notified by the authors prior to survey distribution. Completed forms were then entered in an Excel spreadsheet and SPSS version 10 statistical package (SPSS, Chicago, III) was used. Descriptive data was used in form of frequency tables, which were generated for each question in the survey. An exploratory analysis was conducted to check if advanced stage of training had affected the results. Residents in the first two years of training were compared with residents in their third to fifth year of training. A chi-square test or Fisher Exact test was used to compare proportions were appropriate. A *P* value of less than .05 was considered statistically significant.

## RESULTS

The survey was distributed to 78 surgical residents and 52 forms were retrieved (67%). The response rate was comparable for different participating institutions. Surgical specialties included general surgery (64%) orthopedic surgery (13%), neurosurgery (7%), urology (7%), and plastic surgery (9%). While 45% of residents had comprehensive orientation on admission to the program, only 20% believed it was complete or helpful. Forty-nine percent had an assigned mentor during training. Only 40% of residents felt that their trainers were committed to training, with consultants trained abroad more committed than those trained locally (62% *vs* 36%, *P*=.01). Eighty-eight percent thought that residents should be withdrawn from consultants who are not committed to training. The survey showed that 53% of responding residents were committed to their training, and 75% felt that other hospital staffs were not committed to training programs. Only 38% of respondents were involved in planning their academic activities, and 84% felt that it did not meet their expectations. Only 15% were satisfied with their bedside teaching or operative experience. Only 12% had the objectives of rotations emphasized at the beginning, and although they had enough evaluations (78%), 66% found them low in quality and not helpful. Seventy-eight percent responded that current training does not meet their expectations, but 59% felt it was improving. Eighty-five percent of respondents believed that training abroad was better than local training, and 60% wanted training abroad to be mandatory. Sixty percent of respondents believed that external training should be the sole training method, but 40% thought of it as complimentary to local training. Eighty-seven percent acknowledged that Saudi residents may be more committed to training externally than locally. While 90% felt that training programs should be unified nationally and controlled by one organization, only 6% believed that the current training governing body was effective in monitoring the training. Ninety percent found that there were significant differences in training among different institutions. The effectiveness of the current review of programs by the training council was questioned by 72% of respondents. The exploratory analysis showed that there were no major differences between junior and senior trainees (Table 1).

**Table 1:** Comparison between junior (group I) and senior (group II) residents.

Question	Total (*n*=52)	Group I (*n*=31)	Group II (*n*=21)	*P* value
Was the orientation helpful to shape your training? Yes	20%	19%	27%	0.72
Do you have a constant mentor? Yes	49%	78%	26%	0.02*
Do you believe that consultants are committed to training? Yes	40%	53%	21%	0.04*
Are you involved in planning your academic activities? Yes	38%	47%	25%	0.15
Does local training meet your expectations? Yes	22%	30%	10%	0.16
Were training objectives emphasized to you at the beginning of the program? Yes	12%	14%	10%	1.00
Do you believe that external training is better than local training? Yes	85%	86%	81%	0.69
Do you believe that external training should be mandatory? Yes	60%	66%	53%	0.53
Do you believe that external programs are more committed to training than local ones? Yes	66%	55%	82%	0.11
Do you believe that current training council is effective in monitoring training? Yes	6%	10%	0%	0.27
Do you believe that current reviews by training council are effective in improving programs	28%	40%	10%	0.26

Group I: 1^st^ and 2^nd^ year residents, Group II: 3^rd^, 4^th^, and 5^th^ year residents

* significant

## DISCUSSION

Although medicine involves extensive theoretical preparation by medical schools, postgraduate professional training has a pivotal role in preparing practicing physicians. In particular, surgical training involves an additional facet of training; operative skills. Resident satisfaction with training programs plays a major role in its maintenance. It has been repeatedly cited by surgical programs as the most important reason for not completely filling their training posts.^2^ In Saudi Arabia, surgical training programs have been modified over the last few decades with changes in the monitoring agencies governing those programs. Historically, the Arab Board for Health Specialties has overseen such training for years. In the early 1990s, the Saudi Commission for Health Specialties was founded as a national organization and hence has taken over that task. In addition, there exists few other programs operating in a few training centers with mostly theoretical preparations and examinations toward fellowships awarded pending passage of certain examinations. The latter programs have no practical aspects and are not recognized by most health authorities as the sole training of surgeons. To date, this study is the first and only attempt to explore the satisfaction of Saudi surgical residents with their training. Many factors have been identified in the literature that affect resident satisfaction with training programs. Among them are a balance between education and service,^3^ the quality of attendant's teaching,^4^ interaction with attendants during patient care,^4^ operative experience,^2^,^4^,^5^ and substantial citing of evidence-based literature.^2^

This survey showed that 78% of Saudi surgical trainees were not satisfied with current programs in response to a direct question. This was affirmed by their responses to questions regarding some of the factors shown in the literature to affect residents satisfaction with training programs. Sixty percentage of residents were not satisfied with the faculty's commitment to training, with externally-trained faculties being more committed than locally trained faculties. The latter reflects differences in the attitudes of the trainers shaped by their background training. Faculty motivation to educate residents plays an important role in the dynamics of training programs. It has been shown that there are significant differences among attending faculty members regarding education abilities^6^ and that many trainers' self-assessment differed significantly from that of the residents. In general, trainees believe that they are not getting enough education by faculties.^3^ It was also shown that feedback from trainees could improve the performance of trainers to a significant extent.^7^ On the other hand, many factors have been shown to affect trainers' performance. Among them, time designation for education,^8^ job satisfaction,^8^ motivation for learning,^9^,^10^ recognition and rewards.^11^ From the above, it is obvious that the commitment of faculty members for training and the perception of trainees of such commitment is a complex process and that targeting the above factors to enhance trainers' abilities to educate trainees is essential. In fact, some suggested a comprehensive approach to faculty development,^12^ and its implementation was shown to positively affect the training process.^13^

Operative experience has been shown repeatedly to be one of the most important predictors of satisfaction with surgical training.^2^,^4^,^5^ Our study showed that 85% of surgical trainees were not satisfied with their operative experience. This raises major concerns regarding the quality of current training program. A unique attempt was made to evaluate the operative colorectal surgical experience of advanced general surgical residents at a tertiary care center in Riyadh.^14^ In this study, the surgical experience of Saudi residents was compared with that of surgical residents at a hospital in New Zealand. It was concluded that Saudi general surgical residents were not getting enough colorectal surgical experience despite the fact that the Saudi Board criteria were likely to be met. This study was limited by the fact that it was concerned with only colorectal surgical experience during general surgical training and its results do not reflect the general operative experience in the program. Moreover, the tertiary nature of the Saudi institution with highly advanced cases, and lack of emergency procedures may have skewed the data and led to such a conclusion. Finally, we believe that such a comparison has to be made with multiple training programs with different training methods in order to draw meaningful conclusions. The author used a general term as Western type cases to indicate the type of training needed for residents, but did not discuss the nature of the programs. Western training varies from one country to the other (e.g. North American training differs from European training), but is collectively called Western training. The trainee's attitude and behavior have a major impact on operative experience,^15^ and some felt that residents should “earn” operative experience as a reward for good patient care in the ward.^14^

Operative experience has to be a major component of our programs and new methodologies have to be devised and implemented to overcome current barriers. Possible barriers are trainees' attitude and behavior, faculty commitment, and the availability of a wide spectrum of cases per institution. Some have suggested the use of simulators to practice surgical procedures and adopting an apprenticeship-type model with fewer numbers of faculties per service.^2^

Recently, many medical schools have been founded in Saudi Arabia to produce enough physicians to cover local demands. Currently, only 19.3% of medical doctors are Saudis.^16^ Most of the respondents prefer to be trained at centers abroad and support the notion of making external training mandatory. Training positions locally does not meet the increasing numbers of graduating medical doctors, and moreover, available external training posts are decreasing (personal communication). These facts indicate the need for expedited program reviews and expansion to accommodate the increased numbers of trainees and ensure quality of programs. Moreover, uniformity of training at different institutions is customary to enhance trainees' satisfaction with training.

About 94% of participants believe that the Saudi Council of Health Specialties (national training organization) does not ensure adequate monitoring of the training. This reflects a wide gap between the residents and their training mentors and questions the current communication channels between the two sides. We believe that a transparent relationship coupled by a real wide partnership between the Saudi Council and trainees should be established. Surgical residents deserve a fair representation at the surgical section at the Saudi Council with open channels of communication. This could provide a feedback mechanism that safeguards training and ensures its quality.

In our study, there were no major differences between junior and senior residents regarding responses except in two questions (Table 1). Most junior residents had constant mentors and believed that consultants are committed to training. It seems that this belief changes as trainees advance in the program. The fact that junior and senior residents were not different in responses reflects a general consensus on the results and adds to the validity of the conclusions. Although the exploratory analysis added some comparative data, we acknowledge that the sample size may not convey enough power for this kind of comparison, and thus its results have to be viewed with caution.

This study has a few limitations. The response rate was 67% and one may speculate that nonrespondents may differ from respondents, and thus the results of the study may not be valid. We believe that this response rate is reasonable given the nature of the study and the comparable response rate among institutions. Most of the residents who participated were general surgery trainees (64%). This reflects the fact that most of the surgical residents in training at institutions belong to general surgical programs. Moreover, the questionnaire contained general questions applicable to all surgical disciplines, and it is unlikely that the disciplines have any effect on the results. Our study was conducted in four training centers only, but since these centers are among the largest programs in Riyadh, we feel it is a true representative sample for the rest of the programs.

In conclusion, this data show a general dissatisfaction of surgical residents with their current training programs. It also suggests significant weaknesses of the current program and the ineffectivity of the current monitoring mechanism. We feel that a national review of surgical training programs is warranted in view of these results. These data should be used as a baseline to monitor the effectiveness of interventions applied in the future toward improving surgical training programs.
